# Cost analysis of inguinal hernia repair: the influence of clinical and hernia-specific factors

**DOI:** 10.1007/s10029-021-02372-1

**Published:** 2021-02-08

**Authors:** M. Aydin, P. Fikatas, C. Denecke, J. Pratschke, J. Raakow

**Affiliations:** Department of Surgery, Charité-Universitätsmedizin Berlin, corporate member of Freie Universität Berlin, Humboldt-Universität zu Berlin, and Berlin Institute of Health, Charité Campus Mitte, Campus Virchow Klinikum, Charitéplatz 1, 10117 Berlin, Germany

**Keywords:** Hernia repair, Hernia cost, Cost calculation, Multimorbidity

## Abstract

**Introduction:**

As in the rest of the world, in Germany, inguinal hernia operations are among the most common operations. From an economic standpoint, very little is known about the influence of demographic, clinical or hernia-related parameters on the cost of inguinal hernia repair. We, therefore, evaluated individual patient parameters associated with higher costs with a special focus on multimorbidity.

**Methods:**

A total of 916 patients underwent hernia repair for primary or recurrent inguinal hernia between 2014 and 2017 at a single university center and were included in the analysis. The clinical and financial data of these patients were analyzed to identify cost-increasing parameters.

**Results:**

A majority of patients were male (90.7%), with a mean age of 55 years. The surgical methods utilized were mainly the TAPP (57.2%) and Lichtenstein (41.7%) procedures, with an average duration of surgery of 85 min and an average duration of anesthesia of 155 min. The mean cost of all procedures was 3338.3 € (± 1608.1 €).

Older age, multimorbidity, emergency operations with signs of incarceration, longer hospital stays and postoperative complications were significant cost-driving factors. On the other hand, sex, the side of the hernia (left vs. right) and the presence of recurrent hernias had no influence on the overall direct costs.

**Conclusion:**

From a purely economic point of view, older age and multimorbidity are demographic cost-driving factors that cannot be influenced. The national hospital reimbursement system needs to consider and compensate for these factors. Emergency operations need to be prevented by early elective treatment. Long postoperative stays and postoperative complications need to be prevented by proper preoperative check-ups and accurate treatment.

## Introduction

The basic idea of elective surgical treatment is to cure the disease and provide maximum patient satisfaction with a minimal complication rate. In the current era, however, the economic side of treatment is becoming increasingly important. With rising costs and limited total expenditure in the health care system, to be prepared for the future, it is, therefore, important to develop efficient financing while simultaneously providing high-quality medical services for patients. Therefore, cost analysis is becoming increasingly important for developing solutions to reduce costs.

Around the world, more than 20 million hernias are estimated to be repaired every single year. Accounting for over 200,000 procedures per year, inguinal hernia surgeries are among the most common surgical interventions in Germany, and clinics are faced with large financial challenges [1]. A small economic change regarding a single hernia repair can, therefore, consequently have a large economic outcome on the health care system.

The demographic changes seen with the increasing number of elderly patients who have or want to undergo surgical interventions will make it necessary to adapt surgical care accordingly. In addition to age, the presence of secondary diseases and their combination in terms of multimorbidity also play a significant role. Older, comorbid patients are at higher risk of postoperative complications [2]. However, this group of patients is also associated with significantly higher treatment costs [3, 4]. According to policymakers and health care providers, it has been recognized that more sustainable models of care for multimorbidity should be introduced [5].

From an economic standpoint, very little is known about the influence of demographic, clinical or hernia-related parameters on the cost of inguinal hernia repair. We therefore reviewed our clinical and economic data of patients undergoing inguinal hernia repair with a special focus on multimorbid patients.

## Patients and methods

All patients who underwent surgical treatment for inguinal hernias from the beginning of 2014 to the end of 2017 at the Department of Surgery of the Charité-Universitätsmedizin Berlin at the Campus Charité Mitte and Campus Virchow-Klinikum were examined. Corresponding surgical interventions were retrieved from Charité Berlin Hospital’s information system using the Surgical and Procedural Code (OPS-Code 5-530). Included were elective one-sided or double-sided hernia repairs and emergency surgical interventions. All cases in which the treatment of the hernia was part of a different main surgical procedure were excluded.

In addition to the demographic patient data and surgical data concerning the hernias, main and side diagnoses included in the patient clinical complexity level (PCCL), duration of hospital stay and duration of stay in the extensive care unit were examined. The required personnel expenditure as well as the operative room times (preparation, opening and closure, anesthesia) of the procedures were analyzed. Furthermore, the procedural costs with possible additional costs were evaluated.

Multimorbidity was another factor influencing the cost of the procedures considered. For multimorbidity, we used the definition of the World Health Organization (WHO): the term “multimorbidity” is used throughout to mean the coexistence of two or more chronic diseases in the same individual [5].

The software SPSS, version 22 (IBM, USA) was used for statistical analysis. Categorical variables were compared by means of chi-square tests, and continuous parameters were examined using the Wilcoxon-Mann–Whitney test. The level of significance was defined as *p* < 0.05.

## Results

A total of 916 patients with a mean age of 55 years (17–89 years) underwent elective inguinal hernia surgery. A total of 831 (90.7%) patients were males, and 85 (9.3%) were females. A total of 524 operations (57.2%) were performed laparoscopically with transabdominal preperitoneal (TAPP) procedure, 382 (41.7%) underwent open surgery according to the Lichtenstein technique, and 8 procedures (0.9%) were performed as open surgery according to Shouldice. Half of the patients (52.3%, *n* = 479) complained of a hernia on the right side; in 343 patients (37.4%), the hernia was located on the left, and in 94 patients (10.3%), it was located on both sides. Recurrent hernias were present in 118 patients (12.9%), while incarcerated hernias were present in 44 patients (4.8%). On average, patients spent 2.6 days (1–31 days) in the hospital. There was an average of 2 (0–24) secondary diagnoses, and the PCCL as a measure of the patient clinical complexity level reached a value greater than 0 in 24.9% of the patients (*n* = 228) (Table 1).

**Table 1:** Demographic parameters

	*n* = 916
Gender	
Male	831 (90.7%)
Female	85 (9.3%)
Age [years]	
Mean	55.0 ± 17.1
Median	56 (range 17–89)
Operative procedure	
Open Mesh (Lichtenstein)	382 (41.7%)
Open Suture (Shouldice)	8 (0.9%)
Laparoscopic (TAPP)	524 (57.2%)
Hernia side	
Right	479 (52.3%)
Left	343 (37.4%)
Bilateral	94 (10.3%)
Recurrent hernia [yes]	118 (12.9%)
Emergency/incarceration [yes]	44 (4.8%)
Number of secondary diagnosis	
Mean	3.6 ± 3.8
Median	2 (range 0–26)
PCCL	
0	688 (75.1%)
1	55 (6.0%)
2	57 (6.2%)
3	74 (8.1%)
4	37 (4.0%)
5	5 (0.5%)
Postoperative complications [yes]	38 (4.1%)
Length of hospital stay [days]	
Mean	2.6 ± 2.3
Median	2 (range 1–31)

Values as numbers and percentage or in means ± standard deviation

*TAPP* transabdominal preperitoneal, *PCCL* patient clinical complexity level

The exact operational details and times are displayed in Table 2. The median operative time (incision-suture) of all operations was 85 min (11–401 min). The median time of anesthesia was 155 min. Two percent (*n* = 17) of all patients underwent surgery a second time.

**Table 2:** Operative details

	*n* = 916
Number of operations	
1	898 (98.0%)
2	13 (1.4%)
Number of surgeons	2.0 ± 0.4
Number of scrub nurses	2.0 ± 0.5
Operative set-up time [minutes]	
Mean	40.8 ± 29.4
Median	31 (range 6–98)
Operative time [minutes]	
Mean	92.0 ± 37.7
Median	85 (range 11–401)
Number of anesthetists	1.0 ± 0.0
Number of anesthesia nurses	1.0 ± 0.1
Time of anesthetists [minutes]	
Mean	164.0 ± 48.4
Median	155 (range 74–529)
Time of anesthesia nursing care [minutes]	
Mean	204.2 ± 53.2
Median	195 (range 112–447)

Values as numbers and percentage or in means ± standard deviation

The cost analysis showed a mean cost per patient of 3338.3 € (± 1608.1 €), with a range of 1556–22,721 €. As shown in Table 3, the age of the patient had a remarkable influence on the cost of inguinal hernia repair. All patients over the age of 58 (*n* = 445) generated significantly higher costs (€) than their younger counterparts (3580.4 ± 1868.7 € vs. 3082.1 ± 1226.7 €; *p* < 0.001). The chosen operative procedure showed a significant impact on the cost. The laparoscopic approach (TAPP) was approximately 20€ more expensive than the open procedure (Lichtenstein). Bilateral hernia repair was also more expensive than unilateral repair (one-sided vs. bilateral; 3293.7 ± 1663.2 € vs. 3728.6 ± 921.5 €, respectively; *p* < 0.001). On the other hand, the side of the hernia and the occurrence of a recurrent hernia had no significant impact on the cost of the operation. In the case of incarcerated inguinal hernia surgery, making an emergency operation necessary, the cost was significantly higher (€ 2100) than that for the elective operation (5343.8 ± 3337.9 € vs. 3237.1 ± 1397.5 €, *p* < 0.001). Postoperative complications and related reoperations had a significant cost-increasing impact on cost analysis. Postoperative complications were observed in 38 patients (4.1%). 55.3% of these complications (*n* = 21) were classified as Grade I according to Calvien and Dindo, 15.8% (*n* = 6) as Grade II, 21.1% (*n* = 8) as Grade IIIa and 7.9% (*n* = 3) as Grade IVa. The most common complications were hematoma and postoperative bleeding in 20 cases (52.6%). 11 Patients (1.2%) had to undergo reoperation. The treatment of postoperative complications nearly doubled the cost (6541.9 ± 4777.7 € vs. 3199.7 ± 1127.4 €, *p* < 0.001). In patients with a PCCL above 0, there were significantly greater cost requirements (PCCL = 0 vs. PCCL ≥ 1; 3057.1 ± 808.5 € vs. 4186.9 ± 2735.4 €, respectively, *p* < 0.001). This also applied to patients with a period of hospitalization of more than 2 days compared to patients who stayed less than 2 days (3540.9 ± 1770.0 € vs. 2697.4 ± 565.0 €, *p* < 0.001).

**Table 3:** Influence of demographic and clinical factors on cost of inguinal hernia repair

	Cost [€]	*p*-value
Gender		
Male (*n* = 831)	3321.6 ± 1526.8	0.913
Female (*n* = 84)	3502.0 ± 2259.3	
Age [years]		
< 58 (*n* = 445)	3082.1 ± 1226.7	< 0.001
≥ 58 (*n* = 471)	3580.4 ± 1868.7	
Age [years]		
< 30 (*n* = 90)	3070.5 ± 1043.8	0.066
≥ 30 (*n* = 826)	3367.5 ± 1655.9	
Age [years]		
< 75 (*n* = 789)	3230.2 ± 1380.2	< 0.001
≥ 75 (*n* = 127)	4010.4 ± 2517.2	
Preoperative Diagnostics		
Yes (*n* = 139)	3708.0 ± 1877.4	0.001
No (*n* = 777)	3272.2 ± 1547.1	
Operative procedure		
Lichtenstein (*n* = 383)	3337.8 ± 1818.0	0.011
TAPP (*n* = 525)	3354.7 ± 1447.2	
Side		
Right (*n* = 479)	3357.2 ± 1862.5	0.462
Left (*n* = 343)	3205.1 ± 1333.9	
Side		
One-sided (*n* = 822)	3293.7 ± 1663.2	< 0.001
Bilateral (*n* = 94)	3728.6 ± 921.5	
Recurrent Hernia		
Yes (*n* = 118)	3191.2 ± 1241.2	0.167
No (*n* = 798)	3360.1 ± 1655.0	
Emergency/ Incarceration		
Yes (*n* = 44)	5343.8 ± 3337.9	< 0.001
No (*n* = 872)	3237.1 ± 1397.5	
Additional operative procedure		
Yes (*n* = 134)	4383.6 ± 3088.8	< 0.001
No (*n* = 775)	3156.5 ± 1090.5	
Multimorbidity		
Yes (*n* = 555)	3624.7 ± 1921.5	< 0.001
No (*n* = 361)	2898.1 ± 754.8	
PCCL		
0 (*n* = 688)	3057.1 ± 808.5	< 0.001
≥ 1 (*n* = 228)	4186.9 ± 2735.4	
Postoperative complications		
Yes (*n* = 38)	6541.9 ± 4777.7	< 0.001
No (*n* = 878)	3199.7 ± 1127.4	
Length of hospital stay [days]		
< 2 (*n* = 220)	2697.4 ± 565.0	< 0.001
≥ 2 (*n* = 696)	3540.9 ± 1770.0	

Values as numbers or in means ± standard deviation

*TAPP* transabdominal preperitoneal, *PCCL* patient clinical complexity level

**Fig. 1:**
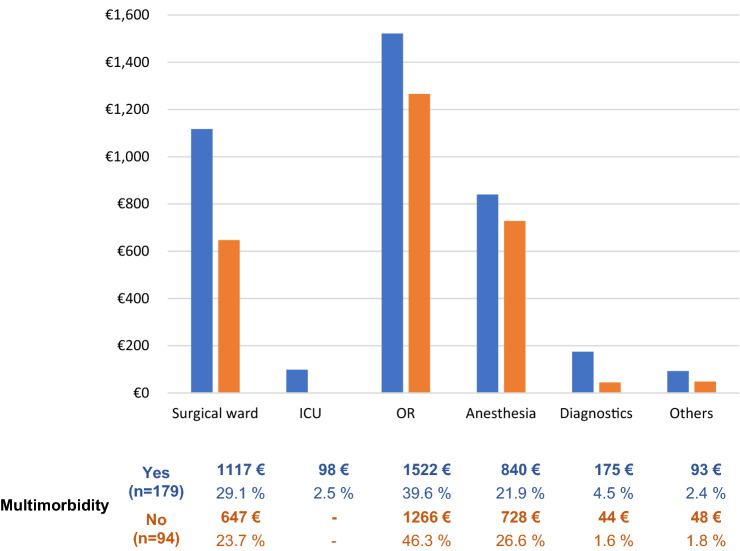
Mean cost distribution in the hospital of all operated patients in 1 year (2019)

In our research, 555 of our patients (60.6%) qualified as multimorbid. As shown in Table 3, the cost for the inguinal hernia operation of multimorbid patients was approximately 750 € more expensive (multimorbidity vs. non-Multimorbidity; 3624.7 ± 1921.5 € vs. 2898.1 ± 754.8 €, respectively, *p* < 0.001). Table 4 shows the influence of multimorbidity on the patient population. There was no difference in the sex distribution on the side of the hernia or regarding the fact that the treated hernia recurred. Multimorbid patients were a mean of approximately 15 years older than patients with fewer secondary diagnoses (60.1 ± 15.0 years vs. 46.1 ± 16.3 years, *p* < 0.001), and their PCCL was significantly higher. Multimorbid patients were approximately four times more likely to present with an incarcerated hernia (6.8% vs. 1.7%, < 0.001), and the operative procedure was significantly more often an open procedure. The operative time for multimorbid patients was almost 10 min longer than the operative time for non-multimorbid patients, with averages of 95.6 min and 86.8 min, respectively (*p* = 0.001). Unfortunately, postoperative complications increased among multimorbid patients. Morbidity rates were 1.1% in the non-multimorbid population and 6.1% in multimorbid patients (*p* < 0.001). Along with the costs, hospital reimbursements were also higher in multimorbid patients (2473.3 ± 716.9 € vs. 3087.0 ± 1772.5 €, *p* < 0.001). Therefore, there was no significant difference in the contribution margin (sum of reimbursement−cost). Figure 1 shows the mean cost distribution in the hospital of all operated multimorbid and healthy patients in 1 year (2019). Expressed as a percentage, the costs for treatment in the surgical ward are higher for multimorbid patients than for patients with fewer secondary diagnoses due to the longer hospital stay.

**Table 4:** Influence of multimorbidity

	Multimorbidity		*p*-value
	No (*n* = 361)	Yes (*n* = 555)	
Gender			
Male	333 (92.2%)	498 (89.7%)	0.244
Female	28 (7.8%)	57 (10.3%)	
Age [years]	46.1 ± 16.3	60.1 ± 15.0	< 0.001
Hernia side			
Right	184 (51.0%)	295 (53.2%)	0.785
Left	140 (38.8%)	203 (36.6%)	
Bilateral	37 (10.2%)	57 (10.3%)	
Recurrent Hernia [yes]	48 (13.3%)	70 (12.6%)	0.783
Emergency/incarceration [yes]	6 (1.7%)	38 (6.8%)	< 0.001
PCCL			
0	357 (98.9%)	331 (59.6%)	< 0.001
1	2 (0.6%)	53 (9.5%)	
2	1 (0.3%)	56 (10.1%)	
3	–	73 (13.2%)	
4	–	37 (6.7%)	
5	–	5 (0.9%)	
Operative procedure			
Open Mesh (Lichtenstein)	121 (33.5%)	262 (47.2%)	< 0.001
Open Suture (Shouldice)	5 (1.4%)	3 (0.5%)	
Laparoscopic (TAPP)	235 (65.1%)	290 (52.3%)	
Operative time [minutes]	86.8 ± 31.6	95.6 ± 40.7	0.001
Postoperative Complications [yes]			
Overall	4 (1.1%)	34 (6.1%)	< 0.001
Hematoma/bleeding	3 (0.8%)	17 (3.1%)	
Electrolyte disorders/dialysis	–	3 (0.5%)	
Wound healing disorders	–	2 (0.4%)	
Early recurrence	–	2 (0.4%)	
Paresthesia	–	2 (0.4%)	
Ileus	–	2 (0.4%)	
Other	1 (0.3%)	6 (1.1%)	
Length of hospital stay [days]	1.9 ± 0.9	3.1 ± 2.8	< 0.001
Overall cost [€]	2898.1 ± 754.8	3624.7 ± 1921.5	< 0.001
Hospital reimbursement [€]	2473.3 ± 716.9	3087.0 ± 1772.5	< 0.001
Reimbursement—cost [€]	− 404.4 ± 869.3	− 515.0 ± 1545.1	0.450

Values as numbers and percentage or in means ± standard deviation

*TAPP* transabdominal preperitoneal, *PCCL* patient clinical complexity level

## Discussion

The aim of the present analysis was to identify fundamental factors that have a direct impact on the economic outcome of inguinal hernia care. Looking more closely at the influence on the economic outcome of individual patients and surgical factors, some conclusions can be drawn from the present study. It was found that the operation was more economical in younger patients than in older patients. After separating the patients into two groups at a median age of 58 years in our population, the younger patients were associated with approximately 500€ greater cost effectiveness than older patients. Interestingly, there was no cost difference between the very young adults (under 30 years) and middle-aged patients (under 58 years). On the other hand, patients older than 75 years had an even more evident influence on the cost of the operation. The fact that the age of the patients was a driver of cost, especially for operations on old patients (over 80 years), which are more expensive, was also found in another analysis from our center in approximately 13,600 surgical cases. Langelotz et al. [4] also found that older patients have a tendency for a longer stay in the hospital, which is one possible explanation for the higher cost associated with older patients, as every day of postoperative inpatient treatment drives cost. Additionally, in our analysis, patients with a hospital stay of over two days were associated with significantly higher costs.

The type of operation had a significant influence on the cost of the treatment, even though the mean difference between open mesh and laparoscopic operations was only approximately 17 €. Multiple other analyses showed higher direct costs for laparoscopic inguinal hernia repair than for the Lichtenstein operation [6–9]. However, this difference decreases when socioeconomic factors are taken into account, making the laparoscopic approach a cost-effective treatment option for inguinal hernia repair [7, 9].

Emergency operations are another cost-driving factor. The average cost for those emergency operations was more than 1.5 times higher than that for the treatment of elective operations. Several other studies had the same conclusion. Gmeiner et al. [10] concluded that emergency surgery in colon diseases increased costs. Shortening the postoperative stay and avoiding emergency operations decrease hospital costs, as morbidity and mortality are clearly increased in comparison to (semi)elective operations [10]. In terms of hernia surgery, Verhels et al. [11] found emergency repair of inguinal hernia in premature infants to be associated with high direct medical costs compared to elective inguinal hernia repair. They concluded that this result needs to be taken into account in the debate on the timing of inguinal hernia repair in infants. In our opinion, this conclusion can be directly transferred to adult patients, and from a purely economic point of view, it needs to be taken into account when talking about watchful waiting, which is clinically justified in patients with asymptomatic or mild symptomatic inguinal hernia [12].

Postoperative complications lead to the greatest cost increase in hernia surgery. If complications occur after inguinal hernia surgery, the overall cost is on average 2.5 times higher than in patients without any complications. This result is also described very clearly elsewhere in the literature [13]. A study from Switzerland on the impact of complications on the costs of major surgical procedures demonstrated the dramatic impact of postoperative complications on full in-hospital costs in an analysis of 1200 procedures. In this study, they analyzed 393 complex hepatobiliary surgeries, 110 major pancreas operations, 389 colon resections, and 308 Roux-en-Y gastric bypasses. Automatic hospital cost and length of stay increased with greater severity and number of complications. They showed that patients with an uneventful course had mean costs per case of $ 27,946. Due to postoperative complications, the costs reached a total of $ 159,345 [14].

Multi-chronic diseases, also called multimorbidities, are faced by health care systems around the world, with many challenges [15]. Age-associated multimorbidity is generally defined as the coexistence of two or more long-term diseases. These patients often have more complicated medical needs than their counterparts with simple diseases. Managing the complexity of multimorbidity requires complex medication regimens. Furthermore, the treatment is more time-consuming, as these patients need to be monitored more frequently by physicians and nurses [16]. As multimorbidity has been such an important issue in surgery in recent years, scientists have conducted an increasing number of studies to obtain deeper knowledge. Epidemiological studies indicate a rapidly rising prevalence of multimorbidity, especially in our Western population. Half of adults in the United states suffer from chronic conditions, and more than two-thirds of elderly individuals have at least two long-term diseases. Within more than a third of patients, at least four different chronic conditions are diagnosed [17]. In Sweden, more than 50% of people between 35 and 75 years of age are reported as multimorbid patients [18]. In the United States, from 2001 to 2010, the presence of multiple chronic conditions (MCCs) increased from 21.8 to 26%. We can see a significant increase in the existence of multimorbidity throughout the recent decades [19]. There are many reasons for this rapid increase in multimorbidity. An aging population, improved diagnosis and detection of diseases and an unhealthy lifestyle, e.g., a high-calorie and salty diet, and little movement associated with the risk of diabetes might be some justifications for this development. Additionally, environmental issues, drug–disease interactions (antidepressants and statins may cause new-onset diabetes) and disease–disease interactions (depression and anxiety can be attributed to cancer diagnosis) might provide an explanation for this growing problem [20]. Especially in hernia surgery, we found that multimorbidity had a very strong influence on our overall cost. Approximately 60% of our patients were considered multimorbid. This unusually high proportion results from the fact that the study was carried out at a university hospital. Generally, the number of multimorbid patients is higher in maximum care hospitals, as the higher complexity of patient care would overwhelm smaller hospitals.

As shown in Table 4, not only the cost for multimorbid patients but also hospital reimbursements were higher. Therefore, the contribution margin as the sum of hospital reimbursement by health insurance minus the cost for the treatment showed no significant difference between the two groups. In the German DRG system, the PCCL is an economic parameter to assess patient complexity and multimorbidity. From an economic point of view, it seems to well reflect patient multimorbidity and associated higher costs and, therefore, the need for higher reimbursements. Since multimorbidity has a significant impact on the treatment cost in surgery, it is mandatory to include this parameter in the hospital reimbursement calculation.

## Conclusion

Several factors influence direct medical costs in patients undergoing inguinal hernia treatment. In particular, cost-driving demographic factors such as age and multimorbidity cannot be directly influenced, and their economic impact should not influence our indications for surgical treatment. It is, therefore, mandatory that the health care system includes these factors in the reimbursement calculation. This will otherwise lead to an undersupply in treatment, as hospitals could refuse operations due to increasing economic pressure.
